# Distinct pathways of straw management practices regulate soil water, salinity, fertility and barley productivity in coastal saline soil

**DOI:** 10.3389/fpls.2026.1969372

**Published:** 2026-09-14

**Authors:** Min Tang, Zhen Lin, Yu Liu, Chao Zhang, Zhijun Jia, Ziang Xie, Huailiang Wang, Lei Xie

**Affiliations:** 1 College of Hydraulic Science and Engineering, Yangzhou University, Yangzhou, Jiangsu, China; 2 Hebei Water Resources Research and Water Conservancy Technology Experimental Promotion Center (Hebei Irrigation Center Experimental Station), Shijiazhuang, Hebei, China

**Keywords:** barley yield, biochar, coastal saline soil, soil moisture, soil salinity, straw management, straw mulching, structural equation modeling

## Abstract

**Introduction:**

Soil salinity severely constrains crop productivity in coastal agricultural regions by impairing soil water availability, nutrient acquisition, and root-zone conditions. Straw-based soil management practices have been widely proposed for saline soil improvement, but their comparative effectiveness and underlying mechanisms remain unclear.

**Methods:**

A two-year container experiment with barley (*Hordeum vulgare* L.) was conducted during the 2023 and 2024 growing seasons to compare the effects of surface straw mulching (SM), straw incorporation (SI), and straw-derived biochar incorporation (BC) with an unamended control (CT) on soil physicochemical properties, crop growth, and yield formation in coastal saline soil.

**Results:**

The results showed that the three straw management practices improved soil–crop performance through distinct pathways. SM exhibited the strongest capacity for regulating soil water and salt dynamics, increasing soil moisture content by 84.9% compared with CT at the heading stage in 2024 and maintaining positive salt migration index values throughout the growing season. Consequently, SM produced the highest grain yield in both years, reaching 6581.95 and 7532.59 kg·ha^-1^ in 2023 and 2024, respectively, representing increases of 6.25% and 19.42% compared with CT. BC showed the greatest potential for improving soil fertility, increasing soil organic matter by more than 33% and enhancing available potassium by 11–26% relative to other treatments in 2023, while maintaining the highest fertility index values (1.55–1.65). SI promoted nutrient accumulation but showed greater interannual variability in soil water and nutrient responses. Structural equation modeling revealed that soil moisture was the most consistent driver of barley yield formation, with its effects primarily mediated through yield components.

**Discussion:**

Overall, surface straw mulching was the most effective strategy for simultaneously improving soil water conservation, salt regulation, and barley productivity, whereas biochar incorporation provided greater benefits for soil fertility improvement. These findings highlight the importance of matching straw management strategies with specific soil constraints to enhance crop production in coastal saline agroecosystems.

## 1 Introduction

Soil salinization is a major constraint on field-crop production because excessive salts reduce soil water potential, disrupt plant nutrient acquisition, and impair soil physical functioning (Pachepsky et al., 2024; Alkharabsheh et al., 2021; Tarolli et al., 2024). Coastal saline soils are particularly susceptible to salt accumulation owing to shallow saline groundwater, strong evaporation, limited natural drainage, and recurrent upward salt transport (Stofberg et al., 2017; Corwin, 2021). These processes deteriorate the root-zone environment and restrict crop establishment, growth, and yield formation. Moreover, climate change and inappropriate land and water management may intensify secondary salinization in many coastal agricultural regions (Gopalakrishnan et al., 2019; Eswar et al., 2021). Developing agronomic practices that simultaneously regulate root-zone water and salt dynamics, improve soil fertility, and sustain crop productivity is therefore essential for the productive use of coastal saline land.

Returning crop straw to the field represents a potentially effective approach for improving salt-affected soils because it supplies organic carbon and can modify soil water movement, nutrient cycling, and structural properties (Zhang et al., 2020; He et al., 2022; Chang et al., 2023). However, the effects depend strongly on the method of application. Surface straw mulching can reduce soil evaporation and restrict the upward movement of salts, thereby improving soil water availability and limiting salt accumulation in the topsoil (Song et al., 2019; Li et al., 2023). Straw incorporation may promote aggregate formation, increase soil porosity and infiltration, and release nutrients during decomposition (Zhang et al., 2023; Latifmanesh et al., 2020). In contrast, straw-derived biochar provides a relatively stable and porous carbon source that can influence soil water retention, nutrient availability, cation exchange capacity, and salt distribution (Huang et al., 2019; Lee et al., 2022; Cui et al., 2022). Nevertheless, biochar responses vary with feedstock, pyrolysis conditions, application rate, and initial soil properties, and its effects on soil pH, electrical conductivity, and crop yield are not consistently positive (Ippolito et al., 2020; Su et al., 2024). Thus, the agronomic effectiveness of these straw management practices cannot be assumed to be equivalent under coastal saline conditions.

Previous studies have documented beneficial effects of individual straw management practices on soil organic matter, water retention, nutrient availability, salinity, and crop yield (Ran et al., 2023; Li et al., 2024; Song et al., 2025). However, direct comparisons of surface mulching, straw incorporation, and straw-derived biochar under the same field and crop-management conditions remain limited. In addition, many studies have focused primarily on individual soil variables, whereas soil water content, salinity, organic matter, and nutrient availability interact to determine crop growth and yield formation. Consequently, the relative importance of these soil properties and the pathways through which different straw management practices affect crop performance remain insufficiently understood. This knowledge gap limits the selection of straw-based practices that are best suited to coastal saline cropping systems.

We hypothesized that the three straw management practices would improve the coastal saline soil–crop system through different mechanisms: surface mulching would predominantly regulate soil water and salt dynamics, whereas straw and biochar incorporation would exert stronger effects on soil fertility and nutrient availability. We further hypothesized that the effects of these practices on crop biomass and yield would be mediated by interacting changes in soil water content, salinity, organic matter, and available nutrients.

To test these hypotheses, a two-year experiment with barley (*Hordeum vulgare* L.) was conducted during the 2023 and 2024 growing seasons using four treatments: no straw amendment as the control (CT), surface straw mulching (SM), straw incorporation into the plough layer (SI), and incorporation of straw-derived biochar (BC). The specific objectives were to: (i) quantify the effects of the treatments on soil water content, salinity, organic matter, and available nitrogen, phosphorus, and potassium; (ii) determine their effects on crop biomass, yield components, and final yield; and (iii) identify the direct and indirect pathways linking straw management, soil physicochemical properties, and crop performance using structural equation modeling. The results are expected to clarify the mechanisms underlying crop responses to alternative straw management practices and provide an agronomic basis for improving crop productivity in coastal saline soils.

## 2 Materials and methods

### 2.1 Experimental site and climatic conditions

A two-season outdoor soil-container experiment was conducted at the Agro-hydrology and Eco-hydrology Experimental Station of Yangzhou University in Yangzhou, Jiangsu Province, China (32°21′N, 119°24′E; 5 m above sea level; **Figure 1**). The experiment encompassed two consecutive barley-growing seasons, from November 2022 to May 2023 and from November 2023 to May 2024.

**Figure 1:**
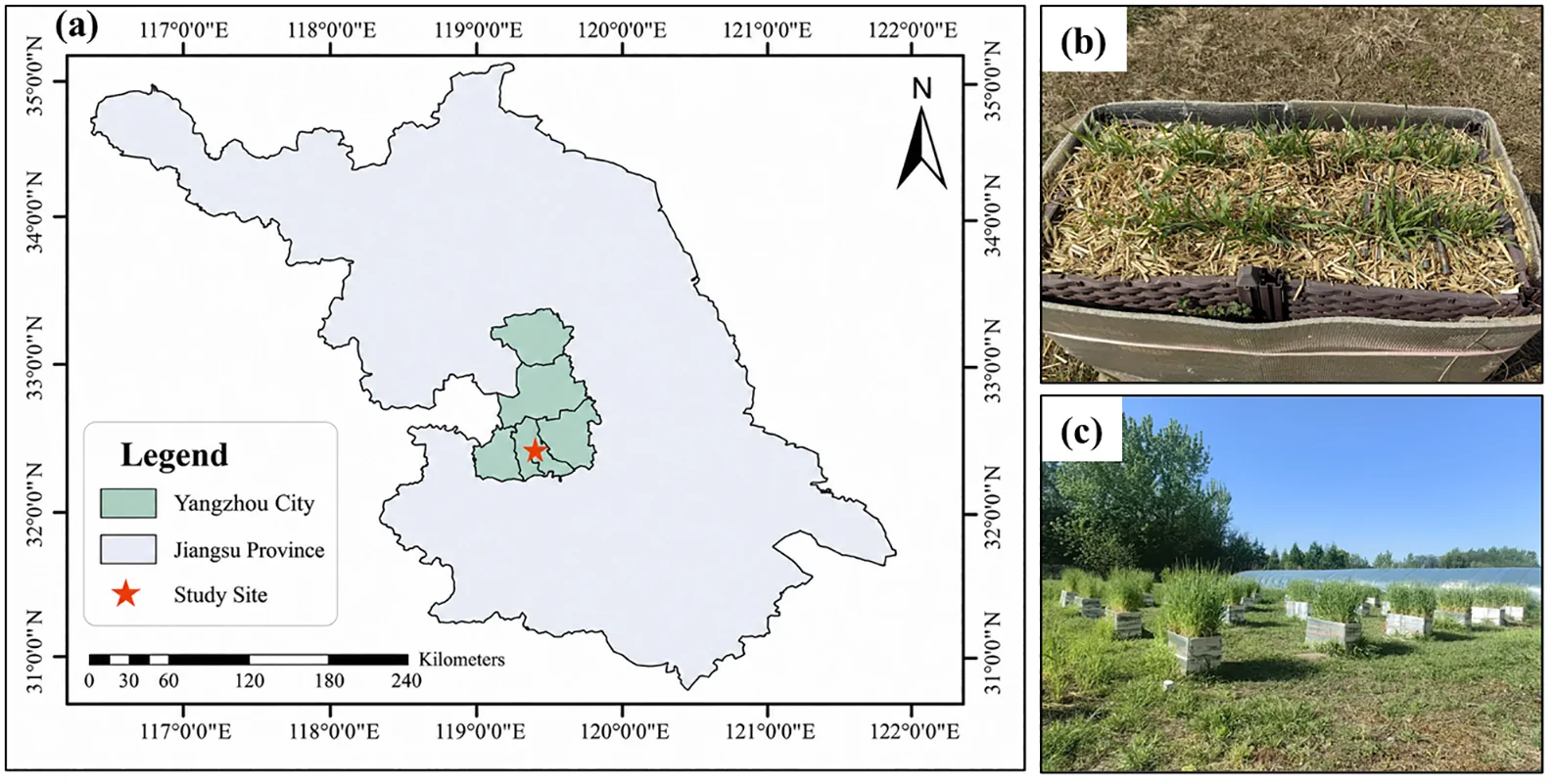
Geographic location of the experimental site and overview of the outdoor soil-container experiment. **(a)** Location of the experimental site in Yangzhou, Jiangsu Province, China; **(b)** barley growth in an experimental container; and **(c)** overall view and spatial arrangement of the experimental containers.

The experimental station is located at the southern margin of the Jianghuai Plain and has a northern subtropical humid monsoon climate. Based on long-term climatic records from 1991 to 2021, the mean annual air temperature is 16.0 °C and the mean annual precipitation is approximately 1217 mm, of which approximately 57% occurs between June and September. January and July are generally the coldest and warmest months, respectively. Daily mean air temperature, precipitation, and irrigation during the two barley-growing seasons are shown in **Figure 2**. Meteorological data during the experimental periods were recorded using an on-site automatic weather station.

**Figure 2:**
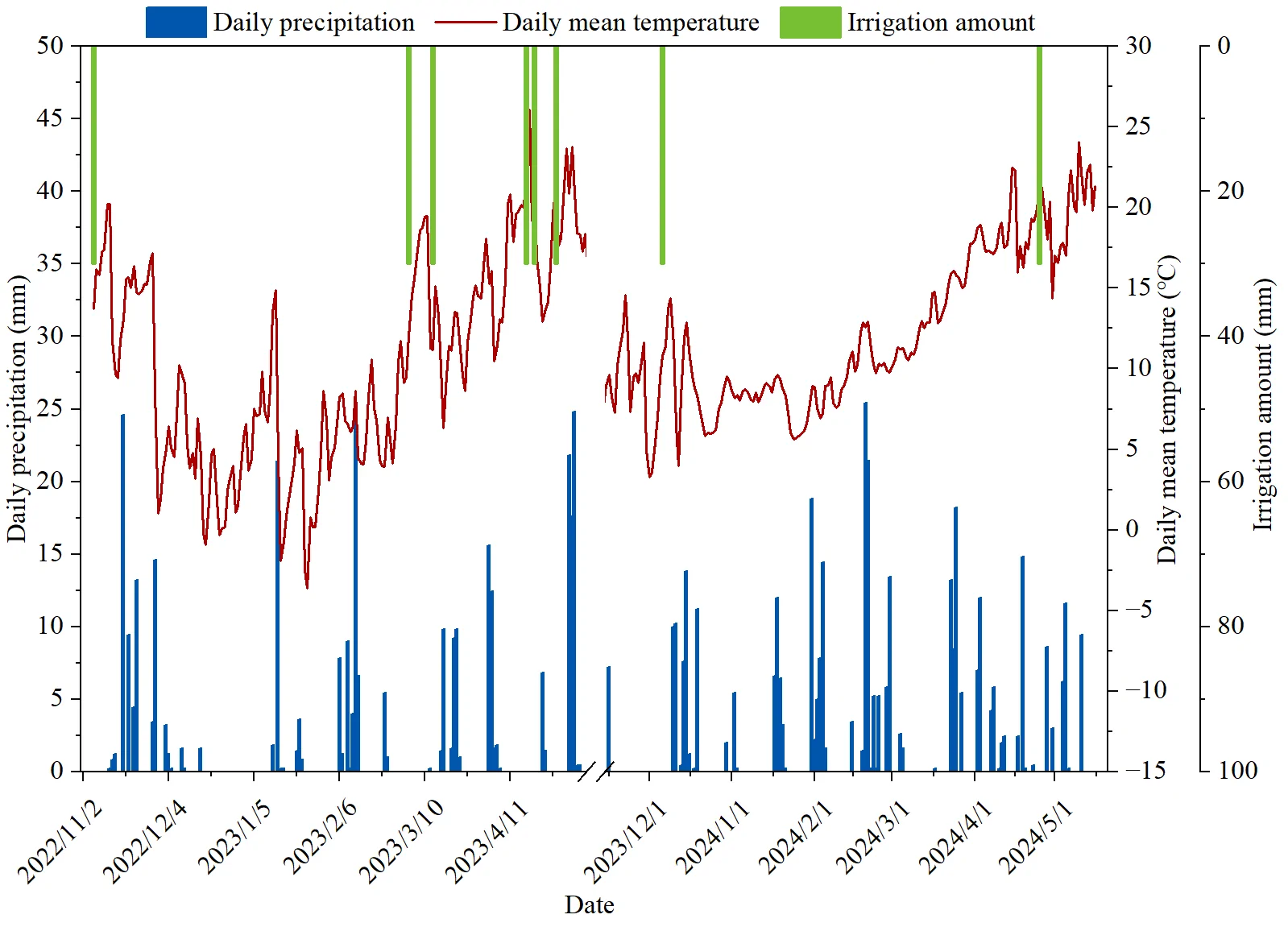
Daily precipitation, mean air temperature, and irrigation events during the experimental period.

### 2.2 Experimental design and crop management

The experiment was conducted in rectangular polypropylene containers measuring 80 cm in length, 40 cm in width, and 52 cm in height. Each container had a surface area of 0.32 m^2^ and was filled with soil to a depth of 40 cm, leaving approximately 12 cm of headspace. A water-collection tray and a removable drainage plug were installed at the base of each container to collect drainage and prevent irrigation overflow. The external walls of the containers were insulated with 6.5-mm-thick aluminum-foil-laminated polyethylene foam to minimize short-term fluctuations in soil temperature.

Coastal saline soil was collected from the 0–40 cm soil layer in the Tiaozini Reclamation Area, Dongtai, Jiangsu Province, China. The soil was air-dried and passed through a 10-mm sieve before packing. The initial total soluble salt concentration was 4.0 g·kg^-1^, corresponding to moderately saline soil. The soil was packed into each container in four successive 10-cm increments. Each increment was uniformly compacted to achieve a target dry bulk density of 1.20 g·cm^-3^, corresponding to an oven-dry soil equivalent of approximately 153.6 kg per container. The same packing procedure was used for all containers.

The experiment comprised four treatments arranged in a completely randomized design with three replicates, resulting in a total of 12 experimental containers. Each container represented one experimental unit, and the 12 containers were randomly positioned within the experimental area. The same treatment structure was maintained in each container throughout the two growing seasons, with the straw and biochar amendments reapplied at the beginning of each growing season. In the first growing season, the amendments were applied on 6 November 2022. For the straw incorporation (SI) and biochar incorporation (BC) treatments, the amendments were incorporated into the upper 0–15 cm soil layer before barley sowing, whereas straw was applied to the soil surface after sowing in the straw mulching (SM) treatment. After the first growing season, the soil was retained in the original containers and was not removed or replaced. Before the second growing season, the SI and BC treatments were reapplied on 14 November 2023 before sowing, while straw was reapplied to the soil surface in the SM treatment after sowing, using the same amendment types and application rates as in the first growing season.

The four treatments were as follows: (1) an unamended control (CT), in which the upper 0–15 cm soil layer was subjected to the same mixing disturbance as in the incorporation treatments but received neither straw nor biochar; (2) SM, in which chopped wheat straw, 3–5 cm in length, was applied uniformly to the soil surface at 0.58 kg·container^-1^, forming a mulch layer approximately 5 cm thick; (3) SI, in which the same amount of chopped wheat straw as used in SM was uniformly incorporated into the upper 0–15 cm soil layer; and (4) BC, in which maize-straw-derived biochar was uniformly incorporated into the upper 0–15 cm soil layer at 0.35 kg·container^-1^. For both growing seasons, the same amendment types and application rates were used for each treatment. The biochar application rate was calculated to provide the same amount of total carbon as the wheat straw applied in the SI treatment. The carbon-equivalent biochar rate was calculated from the measured total carbon concentrations of the wheat straw and biochar according to the following equation: Biochar application rate = straw application rate × straw carbon concentration/biochar carbon concentration.

The wheat straw used in the SM and SI treatments was collected from farmland in the Tiaozini Reclamation Area. Its total nitrogen (N), phosphorus (P), and potassium (K) concentrations were 2.82, 2.23, and 18.94 g·kg^-1^, respectively. Before the experiment, several biochar suppliers were consulted regarding the availability of biochar feedstocks. Maize-straw biochar was selected because it was commercially available and could be obtained promptly at a relatively low cost, whereas wheat-straw biochar generally required customized production with higher costs and a longer preparation period. The biochar was produced from maize straw by pyrolysis at 500 °C for 4 h. It had a pH of 9.0, an ash content of 5.38%, a total N content of 1.55%, and a total carbon (C) content of 72.31%. Before application, the biochar was ground and passed through a 0.15-mm sieve.

Barley (*Hordeum vulgare* L.) was sown on 6 November 2022 and 14 November 2023. Two rows spaced 20 cm apart were established in each container. Seeds were sown at a depth of approximately 3 cm and at a rate of approximately 350 viable seeds·m^-2^, corresponding to approximately 112 seeds per container. After seedling establishment, the plants were thinned to a density of 280 plants·m^-2^, corresponding to approximately 90 plants per container.

Before sowing, a compound fertilizer containing 15% N, 15% P_2_O_5_, and 15% K_2_O was applied as basal fertilizer at 20 g·container^-1^, equivalent to 625 kg·ha^-1^. The fertilizer was uniformly incorporated into the upper 0–15 cm soil layer before sowing. No additional fertilizer was applied during either growing season.

Irrigation was applied when soil water content decreased to approximately 70% of field capacity, and each container was rewatered to approximately 90% of field capacity. The same irrigation thresholds were applied to all treatments. Weeds, pests, and diseases were managed uniformly across treatments in accordance with local agronomic practices.

### 2.3 Measurements and analytical methods

#### 2.3.1 Soil moisture content

During each barley-growing season, soil samples were collected at 10-d intervals from each experimental container using a soil auger. Samples were obtained from the 0–10, 10–20, 20–30, and 30–40 cm soil layers. Fresh soil samples were immediately placed in pre-weighed aluminum tins, sealed to prevent water loss, and weighed. The samples were then oven-dried at 105 ± 2 °C for 24 h or until constant mass. After drying, the tins were sealed, cooled to room temperature in a desiccator, and reweighed. Gravimetric soil moisture content (*θ*, g·g^-1^) was calculated using Equation 1 (Rasheed et al., 2022):

(1) $$\theta =\frac{m_{2}-m_{3}}{m_{3}-m_{1}}\times 100\%$$

where *m*_1_ is the mass of the empty tin (g), *m*_2_ is the combined mass of the tin and fresh soil (g), and *m*_3_ is the combined mass of the tin and oven-dried soil (g).

#### 2.3.2 Soil electrical conductivity

*In situ* soil electrical conductivity (EC) was measured at 10-d intervals concurrently with soil water sampling using a Spectrum EC 450 portable conductivity meter equipped with a direct-insertion soil probe (Spectrum Technologies Inc., Aurora, IL, USA). Measurements were taken at nominal depths of 10, 20, 30, and 40 cm in each container. At each depth, the probe was held stationary until the reading stabilized. The instrument was calibrated and operated according to the manufacturer’s instructions, and EC was recorded in mS·cm^-1^.

#### 2.3.3 Soil chemical analyses

Following barley harvest, soil samples were collected from three evenly distributed locations within each experimental container, avoiding the container walls, plant bases, and previous sampling holes. At each location, soil was collected separately from the 0–10 and 10–20 cm layers. The six subsamples were thoroughly homogenized to obtain one composite sample representing the 0–20 cm soil layer in each container. Visible roots, stones, and other debris were removed, after which the samples were air-dried, ground, and passed through a 0.25-mm sieve. Soil chemical properties were determined using standard analytical procedures (Zhu et al., 2022). Soil organic matter was measured by potassium dichromate oxidation with external heating. Alkali-hydrolysable N was determined by the alkaline hydrolysis diffusion method. Available P was extracted with 0.5 mol·L^-1^ NaHCO_3_ and measured by molybdenum–antimony colorimetry. Available K was extracted with ammonium acetate and determined by flame photometry.

#### 2.3.4 Aboveground dry matter

At the jointing and booting, heading and flowering, and grain filling and maturation stages, the aboveground portions of ten productive tillers were randomly collected from each experimental container. The samples were initially heated at 105 °C for 30 min and subsequently dried in a forced-air oven at 75 °C until constant mass, which generally required approximately 48 h. The oven-dried samples were weighed to determine aboveground dry matter per productive tiller.

#### 2.3.5 Grain yield and yield components

At physiological maturity, all fertile spikes in each experimental container were counted, and spike density was expressed as spikes·m^-2^. Ten spikes were randomly collected from each container and manually threshed to determine the number of grains per spike. All remaining plants in each container were then harvested manually. Grain harvested from each container was air-dried, mechanically threshed, and weighed separately. Grain yield was calculated for each experimental unit and converted to yield per unit ground area. Thousand-grain weight was determined separately for each experimental container. Three 1000-grain subsamples were randomly drawn from the grain harvested from each container and weighed, and their arithmetic mean was used as the thousand-grain weight of that experimental unit.

### 2.4 Derived indices and data processing

#### 2.4.1 Salt migration index

To characterize the vertical redistribution of salts within the 0–40 cm soil profile, a study-specific salt migration index (SMI) was developed based on the vertical distribution of soil EC. For calculation, the soil profile was divided into an upper layer, represented by EC measurements at depths of 10 and 20 cm, and a lower layer, represented by measurements at depths of 30 and 40 cm. The SMI was calculated as the normalized difference between the mean EC of the lower and upper soil layers, as shown in Equation 2:

(2) $$SMI=\frac{\left(EC_{30}+EC_{40}\right)/2-\left(EC_{10}+EC_{20}\right)/2}{\left(EC_{10}+EC_{20}\right)/2}$$

where EC_10_, EC_20_, EC_30_, and EC_40_ represent soil electrical conductivity measured at depths of 10, 20, 30, and 40 cm, respectively. Positive SMI values indicate that EC is greater in the lower soil layer than in the upper layer, representing a vertical salt distribution pattern consistent with downward salt redistribution or leaching. Negative SMI values indicate greater salt accumulation in the upper soil layer and are consistent with upward salt redistribution. An SMI value of 0 indicates no difference in mean EC between the upper and lower soil layers. The magnitude of the SMI reflects the relative degree of vertical differentiation in soil salinity, with larger absolute values indicating a stronger contrast between the upper and lower layers.

#### 2.4.2 Fertility index

To provide an integrated assessment of soil fertility, a study-specific fertility index (FI) was developed based on four key soil fertility indicators: available N, available P, available K, and soil organic matter (SOM). Each indicator was normalized to its corresponding initial soil value before treatment establishment, and the normalized values were integrated using a root-mean-square approach, as described in Equation 3:

(3) $$FI=\sqrt{\frac{{\left(N/N_{0}\right)}^{2}+{\left(P/P_{0}\right)}^{2}+{\left(K/K_{0}\right)}^{2}+{\left(SOM/SOM_{0}\right)}^{2}}{4}}$$

where N, P, K, and SOM represent the measured contents of available N, available P, available K, and soil organic matter, respectively. N_0_, P_0_, K_0_, and SOM_0_ represent the corresponding initial soil values measured before treatment establishment and were 36.6 mg·kg^-1^, 6.5 mg·kg^-1^, 178.0 mg·kg^-1^, and 5.0 g·kg^-1^, respectively. An FI value of 1 therefore represents an overall fertility status equivalent to the initial soil condition, whereas values greater than or less than 1 indicate an overall improvement or decline in soil fertility relative to the initial condition, respectively.

#### 2.4.3 Relative growth rate

Relative growth rate (RGR, g·g^-1^·d^-1^) was used to quantify the relative rate of biomass accumulation per unit of existing plant biomass per unit time. Assuming that biomass accumulation follows an exponential growth pattern, RGR was calculated as the logarithmic increase in dry matter per productive tiller per unit time (Equation 4) (Xiong et al., 2018):

(4) $$RGR=\frac{lnW_{2}-lnW_{1}}{t_{2}-t_{1}}$$

where *W*_1_ and *W*_2_ are the dry matter per productive tiller (g·tiller^-1^) at the initial sampling time *t*_1_ and the subsequent sampling time *t*_2_, respectively, and *t*_2_−*t*_1_ is the interval between the two sampling times (d).

#### 2.4.4 Harvest index

Harvest index (HI, %) was used to quantify the proportion of aboveground biomass allocated to grain yield. It was calculated as the ratio of grain yield to total aboveground biomass (Equation 5) (Rivera-Amado et al., 2019):

(5) $$HI=\frac{grain\;yield}{aboveground\;biomass}\times 100\%$$

#### 2.4.5 Structural equation modeling

Structural equation modeling (SEM) was used to examine the hypothesized relationships among soil properties, biomass accumulation, yield components, and barley grain yield within a physiological chain framework (Kline, 2016). Because all variables included in the model were directly measured rather than represented by multiple observed indicators, the analysis was implemented as an observed-variable path model.

Six soil properties, including soil moisture content, electrical conductivity, available N, available P, available K, and SOM, were specified as exogenous observed variables. Aboveground dry matter accumulation was included as an intermediate variable, whereas grains per productive tiller and thousand-grain weight were included as yield-component variables. Barley grain yield was specified as the final endogenous variable. The hypothesized relationships among these variables were specified *a priori* based on crop physiological mechanisms and previous studies, with particular emphasis on pathways from soil properties to biomass accumulation and yield components and subsequently to grain yield. Covariances among exogenous soil variables were also considered where biologically plausible.

The structural model was expressed as shown in Equation 6:

(6) Y=BY+ΓX+e

where *X* represents the vector of exogenous observed variables, *Y* represents the vector of endogenous observed variables, *B* is the matrix of path coefficients describing the relationships among endogenous variables, *Γ* is the matrix of path coefficients describing the effects of exogenous variables on endogenous variables, and *e* represents the vector of residual errors.

Model development followed five sequential stages. First, the structural relationships among variables were specified *a priori* based on crop physiological mechanisms and previous studies. Particular emphasis was placed on pathways linking soil properties to biomass accumulation and yield components and subsequently to grain yield. Second, model identification was assessed according to the parameter-counting rule. Third, model parameters were estimated using robust maximum likelihood (MLR), which provides robust inference under departures from multivariate normality (Satorra and Bentler, 1994). Fourth, model performance was evaluated using multiple goodness-of-fit indices, including the root mean square error of approximation (RMSEA), comparative fit index (CFI), and Tucker–Lewis index (TLI). Model fit was considered acceptable when RMSEA was below 0.06 and CFI and TLI were above 0.90, with higher values indicating better fit (Hu and Bentler, 1999). Fifth, when key fit indices did not meet these criteria, model modification was guided by modification indices (MIs). Error covariances or additional theoretically justified pathways were considered only when supported by agronomic or physiological reasoning. All model modifications were therefore theory-driven rather than based solely on statistical improvement, thereby reducing the risk of capitalization on chance (MacCallum et al., 1992; Kline, 2016).

### 2.5 Statistical analysis

Data organization and preliminary calculations were performed using Microsoft Excel 2019 (Microsoft Corp., Redmond, WA, USA). Figures were prepared using OriginPro 2021 (OriginLab Corp., Northampton, MA, USA). Statistical analyses were conducted using IBM SPSS Statistics 26.0 (IBM Corp., Armonk, NY, USA). Differences among treatment means were assessed using the Tukey’s test at a significance level of 0.05.

A structural equation model was developed to clarify the pathways through which soil physicochemical properties influenced barley yield components and to quantify the mechanisms by which straw return affected yield formation. Model specification, parameter estimation, and model validation were performed using IBM SPSS Amos 26.0 (IBM Corp., Armonk, NY, USA).

## 3 Results

### 3.1 Changes in profile-averaged soil moisture content

Profile-averaged soil moisture content varied markedly among barley growth stages and treatments in both years (**Figures 3a, b**). The highest soil moisture content occurred during the green-up and tillering stage, ranging from 25.46% to 27.97% in 2023 and from 24.10% to 25.53% in 2024. The SM treatment consistently showed the highest values, reaching 27.97% in 2023 and 25.53% in 2024. Soil moisture content subsequently decreased during the jointing and booting stage, with a particularly pronounced decline in 2023 (**Figure 3a**). Under BC, soil moisture content decreased to 10.05%, representing a 60.6% reduction relative to the green-up and tillering stage, whereas SM maintained a significantly higher value of 19.37% (**Figure 3a**). A further decline was observed during the heading and flowering stage in both years (**Figures 3a, b**). In 2023, soil moisture content under BC decreased to 4.49%, lower than that under CT (6.91%), while SM and SI maintained higher values of 8.44% and 12.09%, respectively (**Figure 3a**). In 2024, soil moisture content under SM reached 13.03% and was significantly higher than those under the other treatments by 5–8 percentage points (*p<* 0.05) (**Figure 3b**). During the grain-filling and maturation stage, soil moisture responses differed among treatments and between years (**Figures 3a, b**). In 2024, soil moisture content under SM remained at 10.30%, whereas in 2023, soil moisture content under SI increased by 79.5% relative to the heading and flowering stage, reaching 21.69% (**Figures 3a, b**). In contrast, soil moisture content under CT declined to 5.64% in 2024. Considerable interannual variation was observed during this stage, with soil moisture content under CT decreasing from 14.37% in 2023 to 5.64% in 2024 (**Figures 3a, b**).

**Figure 3:**
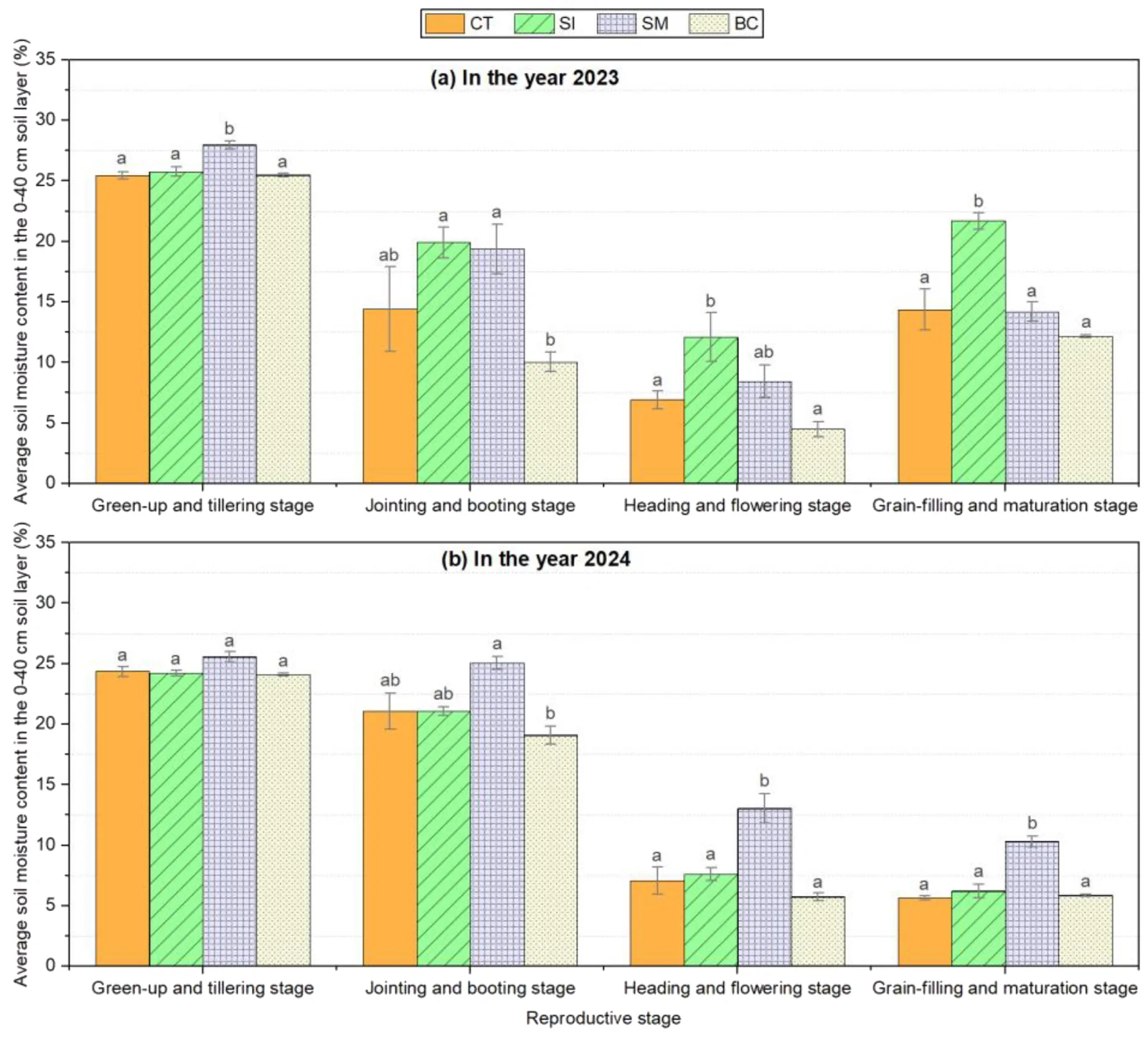
Effects of different straw-returning treatments on average soil moisture content in the 0–40 cm soil layer during barley growth in **(a)** 2023 and **(b)** 2024. Bars represent mean values, and error bars indicate variation among replicates. Different lowercase letters above the bars indicate significant differences among treatments within the same growth stage and year at *p* < 0.05.

Comparisons among treatments showed that SM generally maintained higher soil moisture content during the jointing and booting, and heading and flowering stages (**Figures 3a, b**). In 2024, soil moisture content under SM was 25.04% during the jointing and booting stage, which was 18.9% higher than that under CT. During the heading and flowering stage, soil moisture content under SM reached 13.03%, representing an 84.9% increase compared with CT (*p* < 0.05) (**Figure 3b**). The response of SI varied between years. In 2023, soil moisture content under SI was significantly higher than that under CT during the heading and flowering stage, with values of 12.09% and 6.91%, respectively (*p* < 0.05) (**Figure 3a**). However, in 2024, the difference between SI and CT was much smaller, with corresponding values of 7.60% and 7.05%, respectively (**Figure 3b**). The BC treatment showed the lowest soil moisture contents during the jointing and booting and heading and flowering stages in 2023, with values of 10.05% and 4.49%, respectively (**Figure 3a**). The CT treatment also maintained relatively low soil moisture content during the heading and flowering stage in both years, with values of 6.91% in 2023 and 7.05% in 2024 (**Figures 3a, b**). During the grain-filling and maturation stage, soil moisture content under CT showed strong interannual variation, decreasing from 14.37% in 2023 to 5.64% in 2024 (**Figures 3a, b**).

### 3.2 Dynamics of salt migration

From the green-up and tillering stage to the heading and flowering stage, SMI values remained positive for all treatments in both years, indicating a vertical salt distribution pattern consistent with predominant downward salt redistribution or leaching (**Table 1**). Among the treatments, SM showed the strongest downward salt redistribution pattern in 2023, with an SMI of 2.67 at the green-up and tillering stage. In 2024, BC exhibited the strongest downward salt redistribution pattern, particularly during the jointing and booting stage, when the SMI reached 1.19. By the grain-filling and maturation stage, salt accumulation patterns were observed under some treatments. The CT treatment showed the strongest upward salt redistribution pattern in 2023, with an SMI of −0.56, whereas SI showed slight upward salt redistribution in 2024, with an SMI of −0.03. These changes occurred mainly during the late growing season, when higher temperatures and enhanced evaporation may have favored salt accumulation in the upper soil layers. In contrast, SM and BC maintained positive SMI values during the grain-filling and maturation stage in both years. The BC treatment maintained a positive SMI of 0.40 during this stage in 2024, indicating a salt distribution pattern consistent with continued downward redistribution.

**Table 1:** Salt migration index (SMI) at different growth stages of barley under different experimental treatments in 2023 and 2024.

Year	Treatment	Growth stage			
		Green-up and tillering stage	Jointing and booting stage	Heading and flowering stage	Grain-filling and maturation stage
2023	CT	0.37 ± 0.22a	0.28 ± 0.27a	0.36 ± 0.23a	-0.56 ± 0.62a
	BC	0.53 ± 0.25ac	1.17 ± 0.01ab	1.44 ± 0.40a	0.11 ± 0.06a
	SM	2.67 ± 0.29b	2.17 ± 0.45b	1.08 ± 0.31a	1.31 ± 0.43a
	SI	1.60 ± 0.30bc	0.96 ± 0.52ab	0.64 ± 0.55a	0.68 ± 0.18a
2024	CT	0.94 ± 0.11a	0.93 ± 0.39a	0.56 ± 0.23a	0.22 ± 0.47a
	BC	1.19 ± 0.41a	1.19 ± 0.24a	0.21 ± 0.42a	0.40 ± 0.32a
	SM	1.08 ± 0.21a	0.89 ± 0.24a	0.36 ± 0.21a	0.14 ± 0.10a
	SI	0.72 ± 0.28a	0.83 ± 0.30a	0.17 ± 0.09a	-0.03 ± 0.19a

Values are presented as means ± standard errors (SEM). Different lowercase letters within the same growth stage and year indicate significant differences among treatments (*p* < 0.05).

Overall, SM and BC showed stronger downward salt redistribution patterns than SI and CT (**Table 1**). In 2023, SM maintained positive SMI values above 1.08 throughout all growth stages, indicating a consistent vertical salt distribution pattern toward deeper soil layers. However, the magnitude of SMI under SM decreased in 2024. For example, the SMI of SM at the green-up and tillering stage decreased from 2.67 in 2023 to 1.08 in 2024. In comparison, BC showed relatively stable SMI values between years, particularly during the grain-filling and maturation stage, when positive SMI values were maintained in both years. The response of SI varied between years. Although SI maintained positive SMI values during most growth stages in 2023, it showed a slightly negative SMI during the grain-filling and maturation stage in 2024, with a value of −0.03. The CT treatment showed the greatest tendency toward upward salt redistribution, particularly during the grain-filling and maturation stage in 2023, when the SMI reached −0.56. Although SMI values under CT remained positive in 2024, they were generally lower than those under the straw and biochar treatments.

Across years, SMI values were generally higher in 2023 than in 2024, particularly under SM and BC (**Table 1**). The mean SMI of SM was 1.81 in 2023, whereas the overall intensity of downward salt redistribution decreased in 2024. The SI treatment showed a negative SMI during the grain-filling and maturation stage in 2024, while the SMI of SM at the green-up and tillering stage decreased by approximately 60% between years, from 2.67 in 2023 to 1.08 in 2024. The BC treatment maintained positive SMI values throughout both years and showed relatively stable vertical salt redistribution patterns compared with the other treatments. The positive SMI values observed under CT in 2024 indicated that salt redistribution patterns in unamended soil varied among growth stages and years, although CT showed the greatest tendency toward upper-layer salt accumulation during some periods.

### 3.3 Comprehensive evaluation of soil fertility

The comprehensive evaluation of soil fertility over the two study years showed that all treatments had FI values exceeding 1.35, with clear differences among treatments and between years (**Figure 4d**). The BC treatment showed the highest FI values in both years, reaching 1.65 in 2023 and 1.55 in 2024. This advantage was mainly associated with its relatively high soil organic matter and available K contents. In 2023, soil organic matter content under BC was 15.22 g·kg^-1^, more than 33% higher than those under the other treatments (*p<* 0.05), while available K reached 269.33 mg·kg^-1^, which was 11% to 26% higher than those under the other treatments (**Figures 4c, d**). Although soil organic matter content decreased in 2024, BC maintained the highest value of 7.67 g·kg^-1^. The SI treatment showed an increase in FI from 1.36 in 2023 to 1.51 in 2024. It consistently had the highest available N content, reaching 51.08 mg·kg^-1^ in 2023 and 43.38 mg·kg^-1^ in 2024 (**Figure 4a**). In addition, soil organic matter content under SI reached 6.18 g·kg^-1^ in 2024 and was higher than those under CT and SM (**Figure 4d**). However, available P under SI varied considerably between years. In 2023, its available P content was 5.99 mg·kg^-1^, the lowest among the treatments (**Figure 4b**). These results suggest that the increase in FI under SI was associated with its relatively high available N and soil organic matter contents, whereas the lower available P in 2023 may have constrained the overall increase in FI. The SM treatment showed relatively stable FI values, increasing from 1.38 in 2023 to 1.43 in 2024 (**Figure 4d**). Its main advantage was its relatively high available K content, which reached 242.33 mg·kg^-1^ in 2023 and 246.00 mg·kg^-1^ in 2024; in 2024, available K under SM was the highest among the treatments (*p<* 0.05) (**Figure 4c**). In contrast, SM had relatively low available P in both years, with a value of 13.32 mg·kg^-1^ in 2024, and showed only a limited increase in soil organic matter (**Figures 4b, d**). These differences were reflected in the relatively small increase in FI under SM. The CT treatment showed FI values of 1.35 in 2023 and 1.50 in 2024. Although CT had the highest available P content in 2024, reaching 16.22 mg·kg^-1^ (**Figure 4b**), it generally had lower available N, available K, and soil organic matter contents than the straw and biochar treatments (**Figures 4a, c, d**). Consequently, CT had the lowest FI among the treatments (**Figure 4d**).

**Figure 4:**
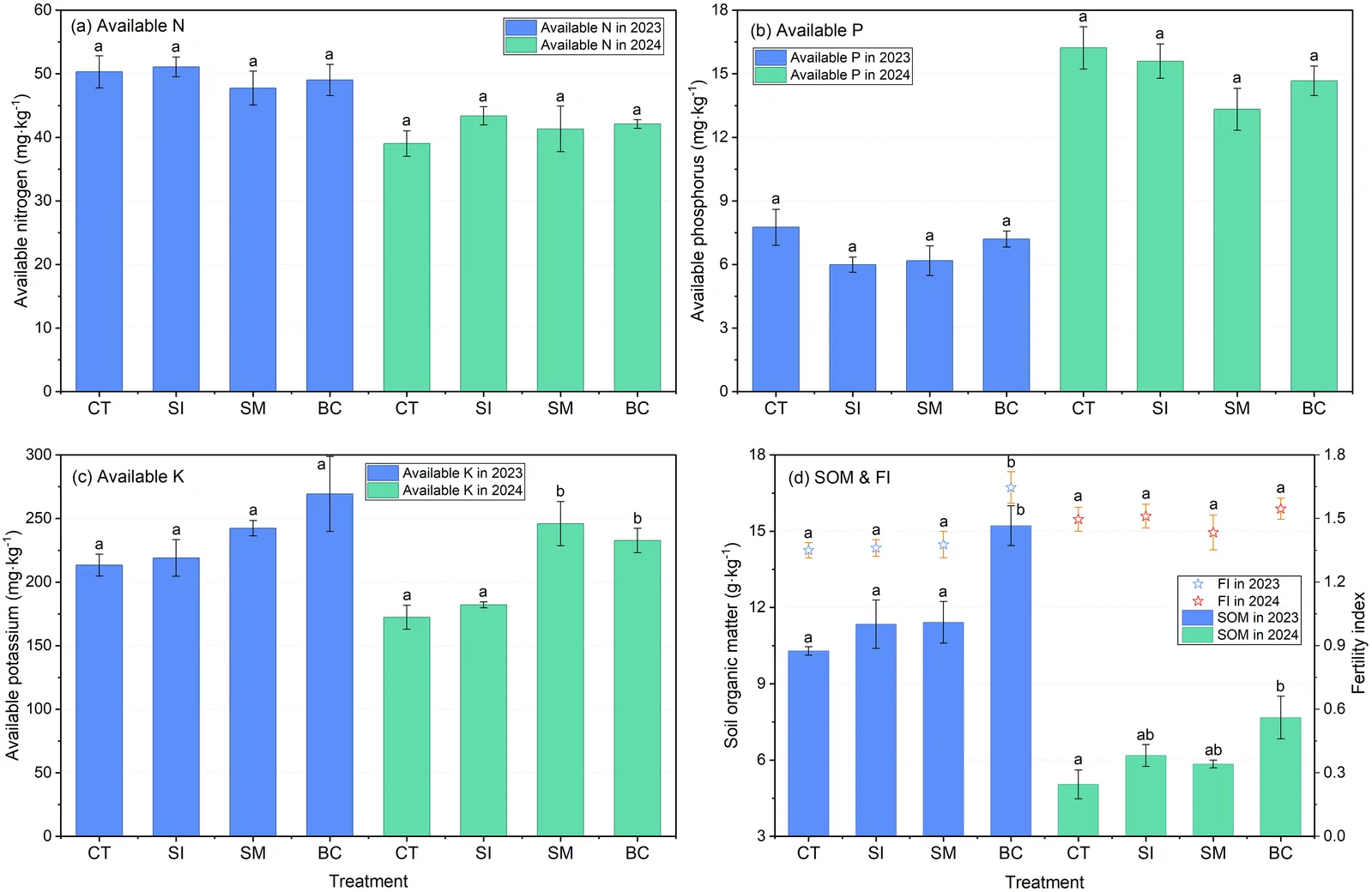
Effects of different straw-returning treatments on **(a)** available N, **(b)** available P, **(c)** available K, and **(d)** SOM and FI following barley harvest in 2023 and 2024. Bars represent mean values, and error bars indicate variation among replicates. Blue and green bars represent data from 2023 and 2024, respectively. In panel **(d)**, pentagram symbols represent the fertility index for each treatment and year. Different lowercase letters indicate significant differences among treatments within the same year at *p* < 0.05.

Soil nutrient contents showed distinct temporal patterns between 2023 and 2024 (**Figures 4a–d**). Soil organic matter content decreased substantially under all treatments, with reductions ranging from 45% to 51% (**Figure 4d**). For example, soil organic matter content under BC decreased from 15.22 g·kg^-1^ in 2023 to 7.67 g·kg^-1^ in 2024. Available N content also decreased by 13% to 22% (**Figure 4a**). Under CT, for example, available N decreased from 50.31 mg·kg^-1^ in 2023 to 39.01 mg·kg^-1^ in 2024. These changes indicated an overall decline in soil organic matter and available N contents between the two years. In contrast, available P content increased markedly by 104% to 160% between the two years (**Figure 4b**). For example, available P under CT increased from 7.75 mg·kg^-1^ in 2023 to 16.22 mg·kg^-1^ in 2024. The increase in available P coincided with the overall increase in FI observed in 2024 (**Figures 4b, d**). All treatments had FI values of at least 1.43 in 2024, whereas BC was the only treatment with an FI above 1.50 in 2023 (**Figure 4d**). The SI treatment reached an FI of 1.51 in 2024, accompanied by its relatively high available N content and an increase in available P to 15.59 mg·kg^-1^ (**Figures 4a, b, d**). In contrast, despite its relatively high available K content, SM showed only a limited increase in FI, from 1.38 in 2023 to 1.43 in 2024. Its available P content was 13.32 mg·kg^-1^ in 2024, the lowest among the treatments in that year (**Figures 4b–d**). The BC treatment maintained relatively stable FI values between years, together with relatively high soil organic matter and available K contents (**Figures 4a, c, d**).

### 3.4 Aboveground dry matter accumulation

Aboveground dry matter accumulation and relative growth rate (RGR) varied considerably between years and among treatments (**Figures 5a–d**). In 2023, aboveground dry matter accumulation at the jointing and booting stage ranged from 1.270 to 1.317 g·tiller^-1^, whereas the corresponding values in 2024 ranged from 0.366 to 0.439 g·tiller^-1^ (**Figures 5a, b**). Biomass accumulation increased substantially from the jointing and booting stage to the heading and flowering stage in both years, although treatment differences varied between years. Relative growth rate also showed distinct temporal patterns among treatments (**Figures 5c, d**).

**Figure 5:**
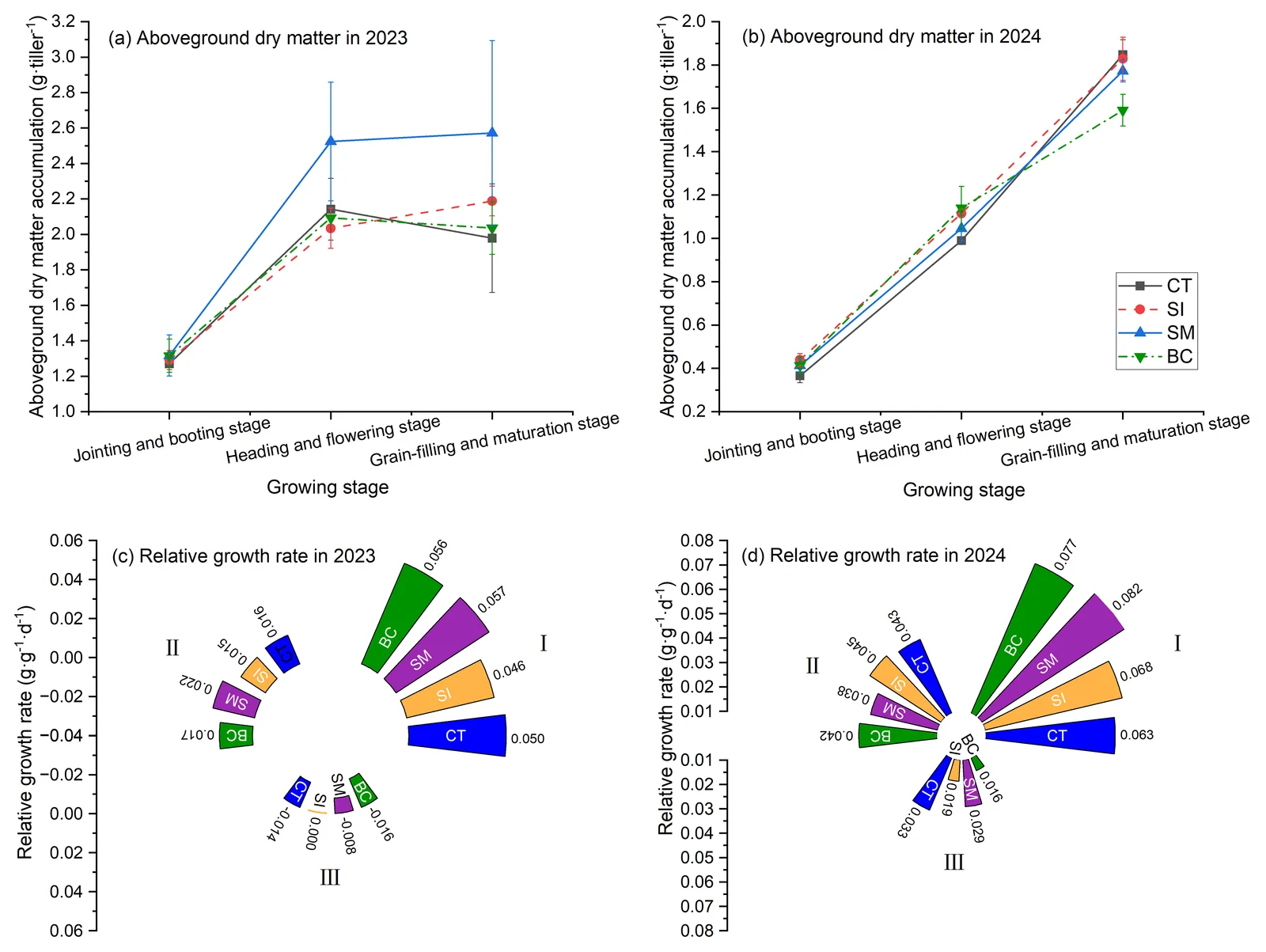
Effects of different straw-returning treatments on aboveground dry matter accumulation and relative growth rate of barley in 2023 and 2024. Panels **(a, b)** show aboveground dry matter accumulation at the jointing and booting, heading and flowering, and grain-filling and maturation stages in 2023 and 2024, respectively. Panels **(c, d)** show the relative growth rates of barley under the four treatments during three successive growth periods (I–III) in 2023 and 2024, respectively. Points and bars represent mean values, and error bars indicate variation among replicates.

The SM treatment showed strong biomass accumulation during the 2023 growing season. Aboveground dry matter accumulation under SM reached 2.523 g·tiller^-1^ at the heading and flowering stage, which was 17.8% higher than that under CT, and subsequently increased to 2.571 g·tiller^-1^ at the grain-filling and maturation stage (**Figure 5a**). The RGR of SM during the corresponding growth period reached 0.0224 g·g^-1^·d^-1^, which was the highest among the treatments in 2023 (**Figure 5c**). However, the biomass response of SM differed in 2024. At the grain-filling and maturation stage, aboveground dry matter accumulation under SM was 1.772 g·tiller^-1^, lower than the 1.848 g·tiller^-1^ recorded under CT (**Figure 5b**). Meanwhile, the RGR of SM during the jointing and booting stage reached 0.0816 g·g^-1^·d^-1^ (**Figure 5d**).

The SI treatment showed relatively consistent biomass accumulation across years. In 2023, aboveground dry matter accumulation under SI reached 2.188 g·tiller^-1^ at the grain-filling and maturation stage, compared with 1.979 g·tiller^-1^ under CT (**Figure 5a**). The RGR under SI during the grain-filling and maturation stage was −0.0003 g·g^-1^·d^-1^, representing the smallest reduction among the treatments (**Figure 5c**). In 2024, aboveground dry matter accumulation under SI reached 1.115 g·tiller^-1^ at the heading and flowering stage and increased to 1.829 g·tiller^-1^ at the grain-filling and maturation stage, which was higher than the 1.591 g·tiller^-1^ recorded under BC (**Figure 5b**).

The BC treatment showed marked interannual variation. In 2023, BC exhibited the largest reduction in RGR during the grain-filling and maturation stage, with a value of −0.0159 g·g^-1^·d^-1^ (**Figure 5c**). In 2024, aboveground dry matter accumulation under BC reached 1.140 g·tiller^-1^ at the heading and flowering stage, while its RGR during the grain-filling and maturation stage was 0.0156 g·g^-1^·d^-1^, which was lower than that under the other treatments (**Figures 5b, d**).

The CT treatment showed contrasting responses between years. In 2023, CT exhibited a negative RGR during the grain-filling and maturation stage (**Figure 5c**). In contrast, CT produced the highest aboveground dry matter accumulation in 2024, reaching 1.848 g·tiller^-1^ at the grain-filling and maturation stage (**Figure 5b**).

### 3.5 Yield and yield components

Yield and yield components differed among treatments and between years (**Figures 6a–d**). In 2023, SM produced the highest grain yield, reaching 6581.95 kg·ha^-1^, which was 6.25% higher than that under CT (**Figure 6c**). The grain number per spike under SM was 30.67, compared with 28.93 under CT, while thousand-grain weight showed little difference between these two treatments, with values of 45.20 and 45.08 g, respectively (**Figures 6a, b**). SI produced the highest thousand-grain weight in 2023, reaching 47.83 g, but its grain yield remained slightly lower than that under CT due to its lower grain number per spike of 27.00 (**Figures 6a–c**). BC produced the lowest grain yield in 2023 at 4797.84 kg·ha^-1^, which was 22.55% lower than that under CT. The harvest index under BC was 50.27%, whereas CT showed the highest harvest index of 67.85% (**Figures 6c, d**). In 2024, SM again produced the highest grain yield, reaching 7532.59 kg·ha^-1^, which was 19.42% higher than that under CT (**Figure 6c**). The grain number per spike and thousand-grain weight under SM were 21.53 and 52.63 g, respectively (**Figures 6a, b**). BC showed higher thousand-grain weight and harvest index in 2024, reaching 50.30 g and 60.60%, respectively (**Figures 6b, d**). Although BC had the lowest grain number per spike at 19.47, its grain yield was 3.47% higher than that under CT (**Figures 6a, c**). Grain yield under SI was 5.66% higher than that under CT, while its harvest index showed considerable variation between years (**Figures 6c, d**).

**Figure 6:**
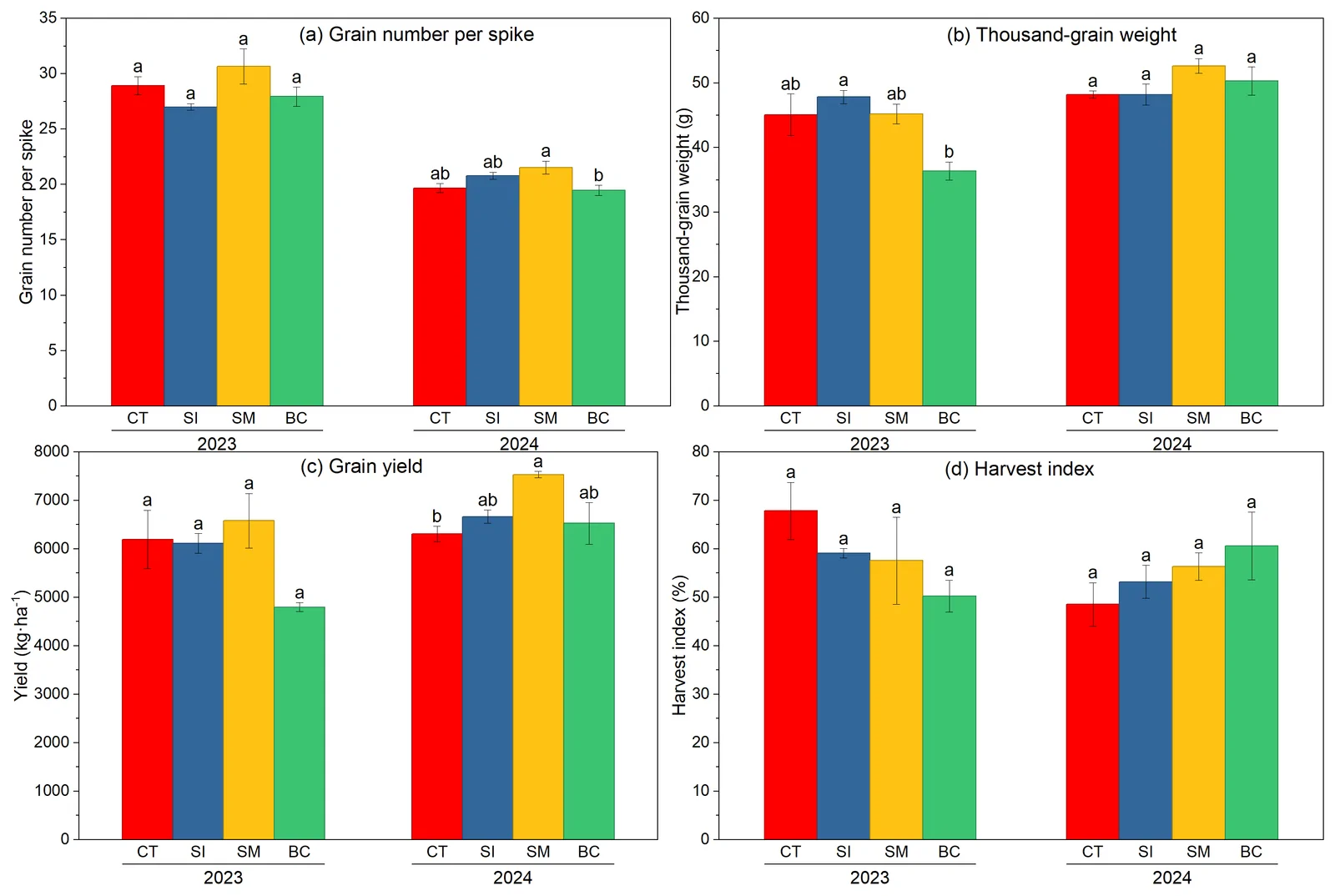
Effects of different straw-returning treatments (CT, SI, SM, and BC) on barley yield characteristics in 2023 and 2024: **(a)** grain number per spike, **(b)** thousand-grain weight, **(c)** grain yield, and **(d)** harvest index. Bars represent mean values, and error bars indicate variation among replicates. Different lowercase letters above the bars indicate significant differences among treatments within the same year at *p* < 0.05.

Clear annual differences were observed in yield components (**Figures 6a, b**). Grain number per spike decreased under all treatments in 2024 compared with 2023, with reductions ranging from 22.96% to 32.03%. For example, grain number per spike under CT decreased from 28.93 in 2023 to 19.67 in 2024. In contrast, thousand-grain weight increased under all treatments. The largest increase occurred under BC, where thousand-grain weight increased by 38.29% to 50.30 g. Under SM, thousand-grain weight increased by 16.44% to 52.63 g (**Figure 6b**). Grain yield and harvest index showed different interannual responses among treatments (**Figures 6c, d**). SM maintained the highest grain yield in both years, with yield increasing by 14.44% in 2024. Grain yield under BC increased by 36.02%, accompanied by an increase in harvest index from 50.27% in 2023 to 60.60% in 2024. In contrast, harvest index under CT decreased from 67.85% in 2023 to 48.55% in 2024 (**Figure 6d**).

The contribution of yield components to grain yield differed between years (**Figures 6a–c**). In 2023, SM had the highest grain number per spike, reaching 30.67. In 2024, grain number per spike under SM decreased by 29.8%, whereas thousand-grain weight increased by 16.4% to 52.63 g. Similarly, thousand-grain weight under BC increased by 38.3% to 50.30 g in 2024 (**Figure 6b**). Although SM produced the highest grain yield in both years, its harvest index was lower than CT in 2023, with values of 57.57% and 67.85%, respectively, and lower than BC in 2024, with values of 56.34% and 60.60%, respectively (**Figures 6c, d**).

### 3.6 Driving factors of barley yield under straw return conditions

The SEM showed satisfactory model performance in both experimental years (**Figure 7**). In 2023, the model fit indices were CFI = 0.972, TLI = 0.939, and RMSEA = 0.0195, while the corresponding values in 2024 were CFI = 0.961, TLI = 0.938, and RMSEA = 0.0165. These values indicated that the hypothesized pathways among soil properties, yield components, and grain yield were adequately supported by the observed data.

**Figure 7:**
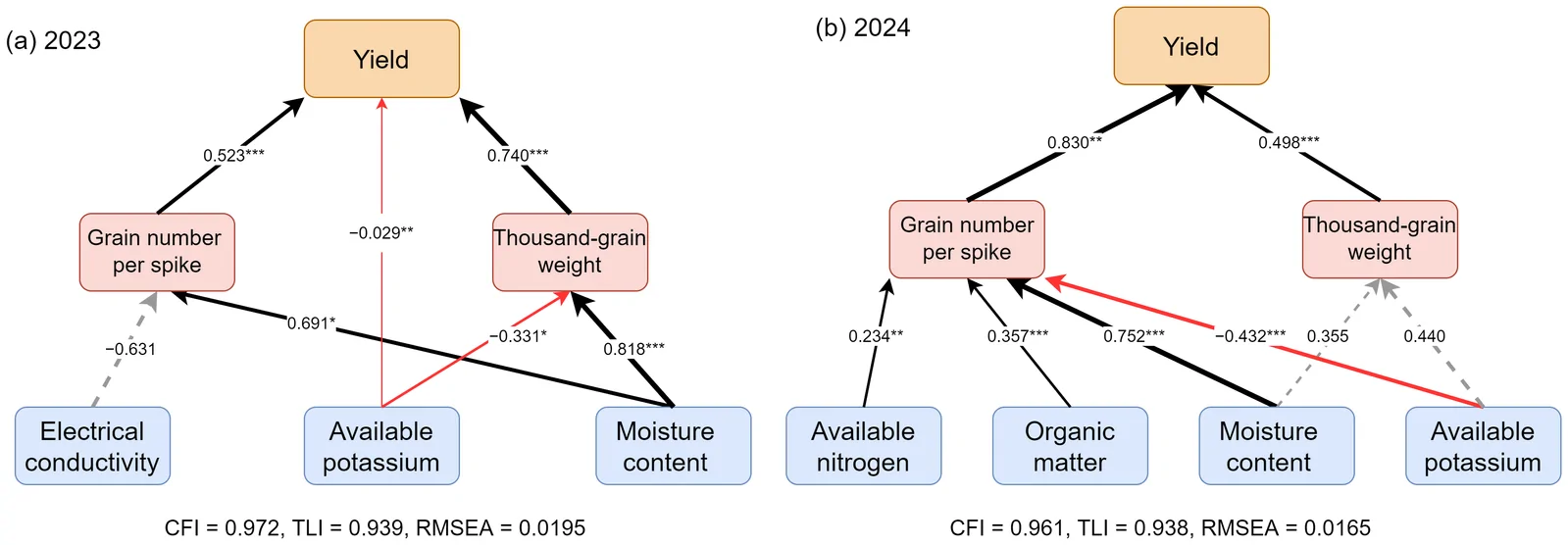
Structural equation models showing the pathways linking soil properties, yield components, and barley yield under straw return conditions. SEM results for **(a)** 2023 and **(b)** 2024. Values on the pathways represent standardized path coefficients. Black solid lines represent significant positive effects, red solid lines represent significant negative effects, and grey dashed lines represent non-significant effects. Line width indicates the magnitude of the standardized path coefficient. Significance levels are indicated by asterisks: *p < 0.05, **p < 0.01, and ***p < 0.001. Model fit indices are shown at the bottom of each panel. CFI, comparative fit index; TLI, Tucker–Lewis index; RMSEA, root mean square error of approximation.

Soil moisture content showed consistent positive relationships with yield components across years (**Figures 7a, b**). In 2023, soil moisture content had positive effects on grain number per spike and thousand-grain weight, with standardized path coefficients of *β* = 0.691 and *β* = 0.818, respectively. In 2024, soil moisture content maintained a positive effect on grain number per spike, with *β* = 0.752, whereas its effect on thousand-grain weight was not significant. The effects of soil nutrient indicators varied between years (**Figures 7a, b**). In 2023, electrical conductivity showed a negative relationship with grain number per spike, with *β* = −0.631. Available potassium showed negative relationships with thousand-grain weight and grain yield, with *β* values of −0.331 and −0.029, respectively. In 2024, available nitrogen and soil organic matter showed positive effects on grain number per spike, with *β* values of 0.234 and 0.357, respectively. Available potassium maintained a negative relationship with grain number per spike (*β* = −0.432), while showing a positive relationship with thousand-grain weight (*β* = 0.440).

The contribution of yield components to grain yield differed between years (**Figures 7a, b**). In 2023, thousand-grain weight showed a stronger positive association with yield than grain number per spike, with path coefficients of *β* = 0.740 and *β* = 0.523, respectively. In 2024, grain number per spike became the dominant yield component, with *β* = 0.830, while the effect of thousand-grain weight decreased to *β* = 0.498.

The total effects of soil indicators further demonstrated annual differences in their associations with grain yield (**Table 2**). Soil moisture content showed the largest positive total effect in both years, with values of 0.967 in 2023 and 0.801 in 2024. Available potassium showed negative total effects in both years, with values of −0.274 in 2023 and −0.140 in 2024. Electrical conductivity had a negative total effect in 2023, with a value of −0.330. In 2024, organic matter and available nitrogen showed positive total effects on grain yield, with values of 0.296 and 0.194, respectively (**Table 2**). All soil indicators affected grain yield mainly through indirect pathways mediated by yield components, while direct effects of soil indicators on grain yield were not observed in the 2024 model (**Table 2**).

**Table 2:** Decomposition of the total, direct, and indirect effects of soil physicochemical properties on barley yield under straw return conditions.

Parameter	2023			2024			
	Moisture content	Electrical conductivity	Available potassium	Moisture content	Available nitrogen	Available potassium	Organic matter
Total effect	0.967	−0.330	−0.274	0.801	0.194	−0.140	0.296
Direct effect	0.000	0.000	−0.029	0.000	0.000	0.000	0.000
Indirect effect	0.967	−0.330	−0.245	0.801	0.194	−0.140	0.296

## 4 Discussion

### 4.1 Mechanisms of straw return in regulating soil moisture dynamics in coastal saline soils

This study demonstrated that straw return regulated soil moisture dynamics in coastal saline soils, with effects varying among management practices, growth stages, and annual climatic conditions. Among the straw return treatments, SM showed the most consistent improvement in soil moisture conservation, particularly during periods of high evaporative demand (**Figures 3a, b**). The higher soil moisture under SM during the early growth stage was mainly associated with the physical barrier created by the straw mulch layer, which reduced direct soil evaporation and improved precipitation storage efficiency (Li et al., 2022). By decreasing evaporative water loss and maintaining higher soil water availability, surface mulching can alleviate early-season water depletion and support crop establishment (Li et al., 2018; Wang et al., 2018). The water conservation advantage of SM was particularly evident during the jointing to heading stages, which are critical periods for barley growth and highly sensitive to water availability. Previous studies have shown that straw mulching can reduce soil evaporation by limiting direct exposure of the soil surface to atmospheric demand and thereby increase water availability in the root zone (Kader et al., 2019; Ramos et al., 2024). In the present study, this effect was more pronounced under the relatively dry conditions of 2023, when SM maintained higher soil moisture during the reproductive transition period (**Figures 3a, b**). These results suggest that surface straw mulching is an effective strategy for buffering seasonal soil water deficits in coastal saline soils.

The effects of SI on soil moisture were more dependent on seasonal water availability. Under the relatively dry conditions in 2023, supplemental irrigation during the heading stage enhanced soil water inputs, and SI maintained higher soil moisture than CT (**Figure 3a**). This response may be attributed to the incorporation of straw residues into the soil matrix, which can improve soil aggregation, increase porosity, and enhance water retention capacity (Zhang et al., 2014; Fu et al., 2021). However, under the wetter conditions in 2024, differences in soil moisture between SI and CT became less pronounced (**Figure 3b**), suggesting that the contribution of straw incorporation to soil moisture regulation may depend on external water supply and background soil moisture conditions.

The BC treatment showed different effects on soil moisture dynamics among growth stages and years. Although soil moisture content under BC was comparable with that under the other treatments during the green-up and tillering stage, it declined markedly during later growth stages under the relatively dry conditions in 2023 (**Figures 3a, b**). Soil moisture content under BC decreased to 10.05% at the jointing and booting stage and further declined to 4.49% at the heading and flowering stage, lower than those under the other treatments (**Figure 3a**). This suggests that the effect of BC on soil water availability depends on soil salinity and seasonal water conditions. Although biochar can enhance soil water retention through its porous structure and high water adsorption capacity, retained water may not always be readily available for plant uptake under high osmotic stress conditions (Razzaghi et al., 2020; Pachepsky et al., 2024).

### 4.2 Dynamic regulation mechanisms of straw return on salt migration in coastal saline alkaline soil profiles

Based on a two-year fixed-site experiment, this study demonstrated that different straw return practices regulated salt distribution within coastal saline alkaline soil profiles by modifying soil water conditions and salt transport processes. The effects of straw return on salt migration varied among growth stages and between years, indicating that soil salinity regulation was closely associated with seasonal water availability and environmental conditions (**Table 1**).

From the green-up and tillering stage to the heading and flowering stage, all treatments showed positive SMI values, indicating a predominant downward salt redistribution pattern within the soil profile (**Table 1**). This pattern was likely associated with increased water inputs from precipitation and irrigation during the early and middle growing seasons, which promoted the downward movement of soluble salts through soil water transport (Zhang et al., 2020; Liu et al., 2022). Straw return further affected salt redistribution by modifying soil water retention and infiltration conditions. The SM treatment reduced surface water loss through soil coverage, whereas BC potentially altered soil water storage characteristics through its porous structure. These effects contributed to enhanced downward salt redistribution, with SM showing the highest SMI value of 2.67 during the green-up and tillering stage in 2023 (**Table 1**).

The effectiveness of straw return treatments in regulating salt migration differed among treatments and years (**Table 1**). In 2023, SM showed the strongest downward salt redistribution pattern, with a mean annual SMI of 1.81. However, the SMI of SM during the green-up and tillering stage decreased by approximately 60% in 2024, suggesting that its salt regulation effect was influenced by annual variations in water conditions and environmental factors (**Table 1**). In contrast, BC showed greater interannual stability, maintaining positive SMI values during the grain-filling and maturation stage in both years. In 2024, BC maintained an SMI of 0.40 during this stage, indicating a relatively stable downward salt redistribution pattern under high evaporative demand (**Table 1**).

The SI treatment showed moderate salt redistribution capacity in 2023 but exhibited slight upward salt redistribution during the grain-filling and maturation stage in 2024, with an SMI of −0.03 (**Table 1**). This variation suggests that the effects of straw incorporation on salt distribution were more sensitive to seasonal environmental changes compared with SM and BC. Overall, SM and BC showed greater potential for maintaining downward salt redistribution, whereas SI exhibited stronger temporal variation in regulating salt distribution within coastal saline alkaline soil profiles (**Table 1**).

### 4.3 Differential regulatory mechanisms of straw return methods on fertility evolution in coastal saline alkaline soils

This study revealed that different straw return methods exerted distinct effects on soil fertility evolution in coastal saline alkaline soils based on a two-year experiment. Among the treatments, BC showed the greatest potential for improving the measured soil fertility indicators, particularly soil organic matter and available potassium (**Figures 4c, d**). The BC treatment maintained the highest FI values in both years, with values of 1.65 in 2023 and 1.55 in 2024 (**Figure 4d**). In 2023, soil organic matter under BC reached 15.22 g·kg^-1^, which was more than 33% higher than that under the other treatments (*p* < 0.05). The improvement observed under BC may reflect the relatively stable carbon characteristics and porous structure of biochar (Han et al., 2020; Shi et al., 2021), although the contribution of its maize-straw feedstock cannot be excluded. Although soil organic matter decreased across all treatments in 2024, BC maintained the highest value of 7.67 g·kg^-1^, suggesting a stronger capacity for preserving soil carbon reserves in coastal saline alkaline soils (**Figure 4d**). Meanwhile, BC consistently maintained higher available potassium contents, reaching 269.33 mg·kg^-1^ in 2023, which was 11% to 26% higher than those under the other treatments (**Figure 4c**).

The SI treatment primarily enhanced soil nitrogen availability. Available N under SI was the highest among all treatments in both years, reaching 51.08 mg·kg^-1^ in 2023 and 43.38 mg·kg^-1^ in 2024 (**Figure 4a**). This response may be attributed to the direct incorporation of straw into the soil, which facilitated residue decomposition and nutrient transformation through closer interactions between straw residues and soil microorganisms (Guan et al., 2020; Chen et al., 2023). Consequently, SI showed an increased FI value in 2024, reaching 1.51 (**Figure 4d**). However, available P under SI showed considerable interannual variation, with a relatively low value of 5.99 mg·kg^-1^ in 2023 (**Figure 4b**), suggesting that phosphorus availability may have constrained the improvement of overall soil fertility during the early stage of straw decomposition.

The SM treatment mainly influenced soil potassium availability. Available K under SM reached 246.00 mg·kg^-1^ in 2024 and was significantly higher than that under the other treatments (**Figure 4c**). However, the increase in soil organic matter was limited, and available P remained relatively low, reaching 13.32 mg·kg^-1^ in 2024 (**Figures 4b, d**). Consequently, FI under SM showed only a moderate increase between years, remaining below 1.45 (**Figure 4d**). These results suggest that straw mulching mainly regulated surface soil nutrient dynamics, whereas improvements in overall soil fertility were limited by insufficient increases in organic matter and phosphorus availability.

One limitation of the present study is that the feedstock origins differed among amendment types. Specifically, the SM and SI treatments used wheat straw, whereas the BC treatment used biochar derived from maize straw. Therefore, the differences observed between BC and the wheat-straw treatments may reflect not only differences in amendment form and pyrolysis but also differences in the original feedstock. Although maize-straw biochar was selected because of its commercial availability, lower cost, and immediate accessibility before the experiment, this difference in feedstock origin should be considered when interpreting the comparative effects of biochar and straw return. Future studies using biochars derived from the same crop residue as the straw treatments would help isolate the effects of feedstock transformation and application form.

### 4.4 Regulatory effects of straw return practices on barley growth and yield in coastal saline alkaline soils

Based on the two-year experiment, this study demonstrated that straw return practices affected barley growth and yield formation through different patterns of biomass accumulation and yield component regulation in coastal saline alkaline soils (**Figures 5a–d**, **6a–d**). The responses of barley to different straw return practices varied between years, indicating that environmental conditions influenced the effectiveness of each management strategy.

Aboveground dry matter accumulation showed strong interannual variation, with biomass accumulation at the jointing and booting stage in 2024 decreasing substantially compared with 2023 (**Figures 5a, b**). This difference was associated with contrasting early-season climatic conditions between years, including lower temperature and reduced precipitation in 2024. Under the relatively favorable conditions in 2023, SM showed the highest RGR during the reproductive growth period, reaching 0.0224 g·g^-1^·d^-1^ (**Figure 5c**). However, under the different environmental conditions in 2024, SM exhibited a higher early-stage RGR but relatively lower biomass accumulation at later stages (**Figure 5d**). In contrast, SI maintained relatively stable dry matter accumulation across years, with limited variation during the grain-filling and maturation stage (**Figures 5a, b**). These results suggest that straw incorporation provided a more stable environment for biomass accumulation under variable seasonal conditions.

Changes in yield components further demonstrated different responses among straw return practices (**Figures 6a, b**). Grain number per spike decreased under all treatments in 2024 compared with 2023, whereas thousand-grain weight increased, indicating a shift in yield formation strategies between years. Under SM, higher grain number contributed to yield formation in 2023, while the increase in thousand-grain weight contributed more strongly in 2024. Under BC, the increase in thousand-grain weight and harvest index in 2024 contributed to improved yield performance compared with 2023 (**Figures 6b, d**). In contrast, CT showed a marked decline in harvest index from 67.85% in 2023 to 48.55% in 2024 (**Figure 6d**).

The relationship between yield and biomass allocation differed among straw return practices (**Figures 6c, d**). Although SM achieved the highest grain yield in both years, its harvest index was lower than that of BC in 2024, with values of 56.34% and 60.60%, respectively. This indicates that higher grain yield was not necessarily associated with higher biomass allocation efficiency under different straw return practices. Overall, straw return methods regulated barley yield formation through different combinations of biomass accumulation, yield component adjustment, and resource allocation strategies in coastal saline alkaline soils.

## 5 Conclusion

Surface straw mulching (SM) provided the most balanced benefits for barley production in coastal saline soils by improving soil water conservation, promoting downward salt redistribution, and maintaining grain yield. During the critical jointing to heading period, SM increased soil moisture content by 84.9% compared with CT at the heading stage in 2024. SM also maintained positive SMI values throughout the growing season, reaching 2.67 at the green-up and tillering stage in 2023. Consequently, SM produced the highest grain yield in both years, reaching 6581.95 kg·ha^-1^ in 2023 and 7532.59 kg·ha^-1^ in 2024, which were 6.25% and 19.42% higher than CT, respectively.

Biochar application (BC) showed the greatest potential for improving soil fertility while maintaining stable salt redistribution patterns. In 2023, soil organic matter under BC was more than 33% higher than that under the other treatments, and available potassium was 11%–26% higher. BC maintained the highest fertility index values in both years, ranging from 1.55 to 1.65, and retained positive SMI values during the grain-filling and maturation stage. Straw incorporation (SI) showed greater interannual variation in soil water and nutrient responses, although it promoted available nitrogen accumulation and improved FI to 1.51 in 2024. In contrast, CT generally showed weaker performance in soil moisture conservation, salt regulation, and yield stability.

The SEM analysis demonstrated that soil moisture was the most consistent factor associated with barley yield formation, with its effects mainly mediated through yield components. The dominant yield component differed between years: thousand-grain weight contributed more strongly to yield formation in 2023 (*β* = 0.74), whereas grain number per spike became the dominant component in 2024 (*β* = 0.83). Overall, SM was the most effective practice for coordinating soil water conservation, salt regulation, and barley productivity under coastal saline soil conditions, whereas BC showed greater potential for improving soil fertility and carbon–nutrient status.

## Data Availability

The original contributions presented in the study are included in the article/supplementary material. Further inquiries can be directed to the corresponding author.
